# Epigenetic silencing of IGFBPL1 promotes esophageal cancer growth by activating PI3K-AKT signaling

**DOI:** 10.1186/s13148-020-0815-x

**Published:** 2020-02-10

**Authors:** Yingge Liu, Meiying Zhang, Tao He, Weili Yang, Lidong Wang, Lirong Zhang, Mingzhou Guo

**Affiliations:** 1 Department of Life Sciences and Technology, Xinxiang Medical University, Jinsui East Road, Xinxiang, 453003 Henan People’s Republic of China; 2 Department of Gastroenterology & Hepatology, Chinese PLA General Hospital, #28 Fuxing Road, Beijing, 100853 People’s Republic of China; 3 Department of Pathology, Characteristic Medical Center of the Chinese People’s Armed Police Force, Tianjin, 300162 People’s Republic of China; 4 State Key Laboratory of Esophageal Cancer Prevention and Treatment, 40 Daxue Road, Zhengzhou, Henan 450052 People’s Republic of China

**Keywords:** IGFBPL1, DNA methylation, Esophageal cancer, PI3K-AKT

## Abstract

**Background:**

There are seven insulin-like growth factor binding proteins (IGFBPs) that bind insulin-like growth factors (IGFs). IGFBP like protein1 (IGFBPL1) is a new member of this family. The function and mechanism of IGFBPL1 in esophageal cancer remains to be elucidated.

**Methods:**

Eight esophageal cancer cell lines, 114 cases of esophageal dysplasia, and 501 cases of primary esophageal cancer samples were examined in this study. Methylation-specific polymerase chain reaction (MSP), immunohistochemistry, Western blot, flow cytometry, RNA interference assay, and xenograft mouse models were employed.

**Results:**

The expression of IGFBPL1was lost and complete methylation was found in KYSE150 and KYSE410 cells. Reduced expression and partial methylation of IGFBPL1 was found in Bic1, KYSE140, KYSE450, KYSE520, and COLO680N cells. High expression and unmethylation was detected in KYSE510 cells. Restoration of IGFBPL1 expression was found in KYSE150 and KYSE410 cells and the expression of IGFBPL1 was increased in Bic1, KYSE140, KYSE450, KYSE520, and COLO680N cells, after 5-AZA-2′-deoxycytidine treatment. IGFBPL1 was methylated in 47.3% (53/114) of esophageal dysplasia and 49.1% (246/501) of human primary esophageal squamous cell carcinoma (ESCC). Methylation of IGFBPL1 was significantly associated with TNM stage (*p* = 0.012), and tumor size (*p* = 0.009). IGFBPL1 inhibited esophageal cancer cell clonal formation and proliferation and induced cell apoptosis and G1/S phase arrest. Further study found that IGFBPL1 is involved in PI3K-AKT signaling and IGFBPL1 suppressed human ESCC xenografts growth in mice.

**Conclusion:**

IGFBPL1 suppresses esophageal cancer cell growth by inhibiting PI3K-AKT signaling in vitro and in vivo. IGFBPL1 is a novel tumor suppressor in human esophageal cancer.

## Introduction

Esophageal cancer is the sixth most common cause of cancer related death worldwide [1]. The prognosis of esophageal cancer remains poor with the overall 5-year survival ranging from 15 to 25% [2, 3]. Esophageal cancer mainly consists of two cell types, esophageal squamous cell carcinoma (ESCC), and adenocarcinoma (EAC). ESCC is the predominant histological type and accounts for 90% of the cases of esophageal carcinoma worldwide [4]. The so-called “Asian esophageal cancer belt,” extending from northern Iran through central China, represents a particularly high-risk area for ESCC, with China alone accounting for more than half of global cases [5].

To identify the genetic differences between ESCC and EAC, Agrawal et al. performed exomic sequencing on 11 cases of EAC and 12 cases of ESCC from the USA. Interestingly, inactivating mutations of NOTCH1 were identified in 21% of ESCCs but not in EACs. Notably, NOTCH1 mutations were more frequent in North American ESCCs (11 of 53) compared to ESCCs from China (1 of 48 cases) according to Sanger sequencing validation [6]. Using whole genomic sequencing of ESCC samples from China, Song et al. found that somatic aberrations are mainly involved in the Notch, Wnt, and cell cycle pathways [7]. In addition to NOTCH1 mutations, Hu et al. revealed frequent mutations in PIK3CA and PTEN in ESCC [8].

Epigenetics stands at the interface of the genome, development, and environmental exposure [9]. DNA methylation is the most useful epigenetic marker for human disease studies because it is stable [10]. A great deal of epidemiologic evidence supports a relationship between dietary exposure in early life and long-term health [11]. Moreover, diet can cause profound changes in the epigenome, leading to human disease [9]. Aberrant DNA methylation has been reported in genes involved in cell cycle, DNA damage repair, Wnt, TGF-β, and NF-κB pathways [12, 13].

The insulin-like growth factor binding proteins (IGFBPs) bind to insulin-like growth factors (IGFs) and regulate their functions. Based on differential affinities to insulin growth factors (IGFs), IGFBPs are classified into two groups: IGF high-affinity binding proteins (IGFBP1-6) and IGF low-affinity binding proteins, such as IGFBP-related proteins (IGFBP-Rp1-10). IGFBP like protein1 (IGFBPL1) is another member of IGFBPs family [14, 15]. IGFBPL1 was reported to be involved in neural development through PI3K signaling [16]. The expression of IGFBPL1 was reduced in breast cancer, and its expression was regulated by promoter region methylation [17]. The expression and function of IGFBPL1 in esophageal cancer remain to be elucidated.

## Materials and methods

### Human tissue samples and cell lines

A total of 501 cases of ESCC, 114 cases of esophageal dysplasia, and 5 cases of normal esophageal mucosal were collected from the Chinese PLA General Hospital in Beijing. The collection of all samples was based on the approval and guidelines of the Institutional Review Board of the Chinese PLA General Hospital. Among ESCC patients, 316 cases were male and 185 cases were female. The median age was 63 years old (range 41–87 years old). All cancer samples were classified according to the TNM staging system (AJCC2018), including tumor stage I (*n* = 30), stage II (*n* = 112), stage III (*n* = 333), and stage IV (*n* = 16). Eight esophageal cancer cell lines were employed in this study, including Bic1, KYSE140, KYSE410, KYSE450, KYSE150, KYSE520, KYSE510, and COLO680N cells. All cell lines were previously established from primary esophageal cancer and maintained in 90% RPMI media 1640 (Invitrogen, CA, USA) supplemented with 10% fetal bovine serum. Cells were passaged 1:3 when total confluence (~ 10^6^cells) was reached in a 75 cm^2^ culture flask (NEST Biotechnology, Jiangsu, China).

### 5-AZA-2′-deoxycytidine treatment

The esophageal cancer cell lines were seeded in 10 cm^2^ culture dishes (30% confluency) 12 h before treatment with 2 M of 5-AZA-2′-deoxycytidine (5-AZA, Sigma, MO, USA). RPM1640 medium containing 5-AZA-2′-deoxycytidine was changed every 24 h for a total of 96 h.

### RNA isolation and semi-quantitative RT-PCR

Total RNA was extracted using Trizol Reagent (Life Technologies, Carlsbad, CA, USA), quantified by spectrophotometer and qualified by agarose gel electrophoresis.

First-strand cDNA was synthesized according to the manufacturer’s instructions (Invitrogen, Carlsbad, CA, USA). A total of 5 μg RNA was used to synthesize the first-strand cDNA and diluted to 100 μl. Subsequently, 2 μl of diluted cDNA mixture was used for PCR amplification in a final 25-μl reaction volume. PCR primer sequences for IGFBPL1 were as follows: 5′-GTGAGGGCTGTGCCTACCC-3′ (F), 5′-CATCA CATGCGGTCATCGGG-3′ (R). PCR amplification was for 35 cycles, and the length of products is 308 bp. The primers for GAPDH were as follows: 5′-GACCACAGTCCATGCCATCAC-3′ (F), and 5′-GTCCACCACCCTGTTGCTG TA-3′ (R). As an internal control, GAPDH was amplified for 25 cycles. The amplified PCR products were examined by 2% agarose gels.

### DNA extraction, bisulfite modification, methylation-specific PCR, and bisulfite sequencing

DNA was prepared by the proteinase K method. Bisulfite treatment was performed as previously described [18, 19]. Methylation-specific PCR (MSP) primers were designed according to genomic sequences around transcriptional start sites (TSS) and synthesized to detect unmethylated (U) and methylated (M) alleles. Bisulfite sequencing (BSSQ) was performed as previously described [20]. BSSQ products were amplified by primers flanking the targeted regions including MSP products. MSP primers were as follows: 5′-TGTAGGTTCGGTTAATTAGCGGTCGC-3′ (MF) and 5′-GAAACAACGACGACGCCTCTACTTCG-3′ (MR); 5′-GGTGTAGGTTTGGTT AATTAGTGGTTGT-3′ (UF) and 5′-CCAAAACAACAACAACACCTCTACTTA-3′ (UR). BSSQ primers were as follows: 5′-TYGGGTTGGAGTAGYGGTT-3′ (F) and 5′-AAACRCCRAACAACCCTCTAA-3′ (R). MSP amplification conditions were as follows: 95 °C 5 min, 1 cycle; 95 °C 30 s, 60 °C 30 s, and 72 °C 40 s, 35 cycles; and 72 °C 5 min, 1 cycle. BSSQ PCR amplification conditions were as follows: 95 °C 5 min, 1 cycle; 95 °C 30 s, 64 °C 30 s, and 72 °C 45 s, 3 cycles; 95 °C 30 s, 61 °C 30 s, and 72 °C 45 s, 3 cycles; 95 °C 30 s, 58 °C 30 s, and 72 °C 45 s, 3 cycles; 95 °C 30 s, 55 °C 30 s, and 72 °C 45 s, 26 cycles; and 72 °C 5 min, 1 cycle.

### Construction of lentiviral IGFBPL1 expression vector and screening of stably expressing cell lines

Human full-length IGFBPL1 coding DNA sequence (GenBank accession number: NM_001007563) was cloned into the pCDH-CMV-MCS-puro vector. The primers used were 5′-GCCACCATGCCGCGCTTGTCTCTGCTC-3′ (F) and 5′-CGCTCGAGTCACATGCGGTCATCGGGAG-3′(R). The HEK-293 T cell line was maintained in DMEM (Invitrogen, CA, USA) supplemented with 10% fetal bovine serum. IGFBPL1 expressing lentiviral vector was transfected into HEK-293 T cells (5 × 10^6^ per 100-mm dish) using Lipofectamine 3000 Reagent (Invitrogen, CA, USA) at a ratio of 1:3 (DNA mass: Lipo mass). Lentivirus was added to the growing medium of KYSE150 and KYSE410 cells, and IGFBPL1 stably expressed cells were selected by puromycin at a concentration of 2 μg/ml (KYSE150) or 1.5 μg/ml (KYSE410) for 3 days.

### RNA interference assay

Selected siRNAs targeting IGFBPL1 and the RNAi negative control duplex were used in this study. RNAi negative control duplex: 5′-UUCUCCGAACGUGUCACGUTT-3′ (F); 5′-ACGUGACACGUCGGAGAATT-3′ (R); siIGFBPL1-847: 5′-GCUCCCGAUGACCGCAUGUTT-3′ (F); 5′-ACAU GCGGUCAUCGGGAGCTT-3′ (R); siIGFBPL1-482: 5′-GCGAGUUCGCUCCUG UGGUTT-3′ (F); 5′-ACCACAGGAGCGAACUCGCTT-3′ (R). The RNAi oligonucleotide and RNAi negative control duplex were transfected into KYSE510 cells, which expressed high levels of IGFBPL1.

### Colony formation assay

IGFBPL1 unexpressed and stably expressed cells (KYSE150 and KYSE410) were seeded at 400 cells per well in 6-well culture plates in triplicate. Before and after the knockdown of IGFBPL1, KYSE510 cells were seeded in 6-well plates at a density of 400 cells per well. After 12 days, cells were fixed with 75% ethanol for 30 min and stained with 0.2% crystal violet. The number of clones was then counted. Each experiment was repeated three times.

### Cell viability assay

Cells were plated into 96-well plates at a density of 2.5 × 10^3^ cells per well, and cell viability was measured by the methyl thiazolyl tetrazolium (MTT) assay (KeyGEN Biotech, Nanjing, China) at 0, 24, 48, and 72 h. Absorbance was measured on a microplate reader (Thermo Multiskan MK3 [Thermo Fisher Scientific, Danvers, MA, USA]) at a wavelength of 490 nm.

### Flow cytometry

IGFBPL1 unexpressed and re-expressed KYSE150 and KYSE410 cells were starved for 12 h and then stimulated with 10% FBS for 24 h. Cells were fixed with 70% ethanol and treated using the Cell Cycle Detection Kit (KeyGEN Biotech). The cells were then analyzed by a FACS Caliber flow cytometer (BD Biosciences, Franklin Lakes, NJ, USA). The same method was used to analyze KYSE510 cells before and after IGFBPL1 knockdown. Cell phase distribution was analyzed using MODFIT software (Verity Software House, ME, USA). KYSE150 and KYSE410 cells were transiently transfected with pcDNA3.1+ and pcDNA3.1+ IGFBPL1 vectors. Cell apoptosis was analyzed by FITC Annexin V assay kit according to the manufacturer’s instructions (BD Bioscience, Franklin). Apoptosis of KYSE510 cells with or without knockdown of IGFBPL1 was also analyzed. Each experiment was repeated three times.

### Western blot

Proteins from esophageal cancer cells were collected and Western blot was performed as described previously [21]. Antibodies were diluted according to the manufacturer’s instructions. Antibodies were as follows: IGFBPL1 (RD,MN,US); Cleaved caspase-3 (Cell Signaling Technology, Danfoss, MA, USA); BCL2 (Cat: 12789-1-AP, Proteintech, USA); CyclinE1 (Cat: 11554-1-AP, Proteintech, USA); CyclinA2 (Cat: 18202-1-AP, Proteintech, USA); CyclinD1 (Cat: 60186-lg, Proteintech,USA); AKT (Cat: 60203-2-lg, Proteintech, USA); p-AKT (Cat: 66444-1-lg, Proteintech, USA); mTOR (Cat: 2983S, Cell signaling technology); p-mTOR (HuaXingBoChuang, China); PI3K (Cat: 20584-1-AP, Proteintech, USA); MYC (Cat: 10828-1-Ap, Proteintech, USA), and β-actin (Cat: AF0003, Beyotime Biotech, Jiangsu, China).

### Immunohistochemistry

Immunohistochemistry (IHC) analysis was performed in human ESCC samples and paired adjacent tissue samples. The IGFBPL1 antibody was diluted to 1:400 (CAT: bs15569R, BIOSS), PI3K antibody was diluted to 1:400 (Cat: 20584-1-AP, Proteintech, USA), p-mTOR antibody was diluted to 1:200 (HuaXingBoChuang, China), and p-AKT antibody was diluted to 1:200 (Cat: 66444-1-lg, Proteintech, USA). The staining intensity and range of the stained areas were scored using the German semi-quantitative scoring system as previously described [20, 22, 23]. The staining intensity of IGFBPL1 expression was quantified as follows: no staining = 0, weak staining = 1, moderate staining = 2, and strong staining = 3; the extent of staining was defined as follows: 0% = 0, 1–24% = 1, 25–49% = 2, 50–74% = 3, and 75–100% = 4. The final immune response score (0–12) was determined by multiplying the intensity score by the staining score.

### Xenograft mouse model

The unexpressed and stably expressed KYSE150 cells (4 × 10^6^ cells in 0.15 ml phosphate buffer) were injected subcutaneously into the right dorsal side of female Balb/c nude mice of 4 weeks old (*n* = 5). Tumor volume was measured every 4 days for 24 days starting 4 days after implantation. The tumor volume was calculated according to the *V* = *L* × *W*^2^/2 formula, where *V* represents the volume (mm^3^), *L* represents the largest diameter (mm), and *W* represents the minimum diameter (mm). All procedures were approved by the Animal Ethics Committee of the Chinese PLA General Hospital.

### Data analysis

RNA-Seq data for expression of IGFBPL1 in the esophageal cancer tissue dataset were downloaded from the Tumor Genome Atlas (TCGA) (http://xena.ucsc.edu/, 04/6/2019). IGFBP1-6 RNA expression data were extracted from the Tumor Genome Atlas (TCGA) dataset for esophageal cancer tissue samples and IGFBP1-6 RNA expression data for normal esophagus were extracted from GTEx (http://xena.ucsc.edu/, 21/12/2019). Statistical analysis was performed using SPSS 17.0 software (SPSS, Chicago, IL, USA). All data are presented as means plus or minus SD and analyzed using the Student’s *t* test. The chi-square test and the Fisher’s exact test were used to analyze the association between IGFBPL1 methylation status and clinicopathologic factors, as well as the association between IGFBPL1 expression and methylation status. A *p* < 0.05 was considered to be statistically significant.

## Results

### The expression of IGFBPL1 is downregulated by promoter region methylation in esophageal cancer cell lines

**Fig. 1:**
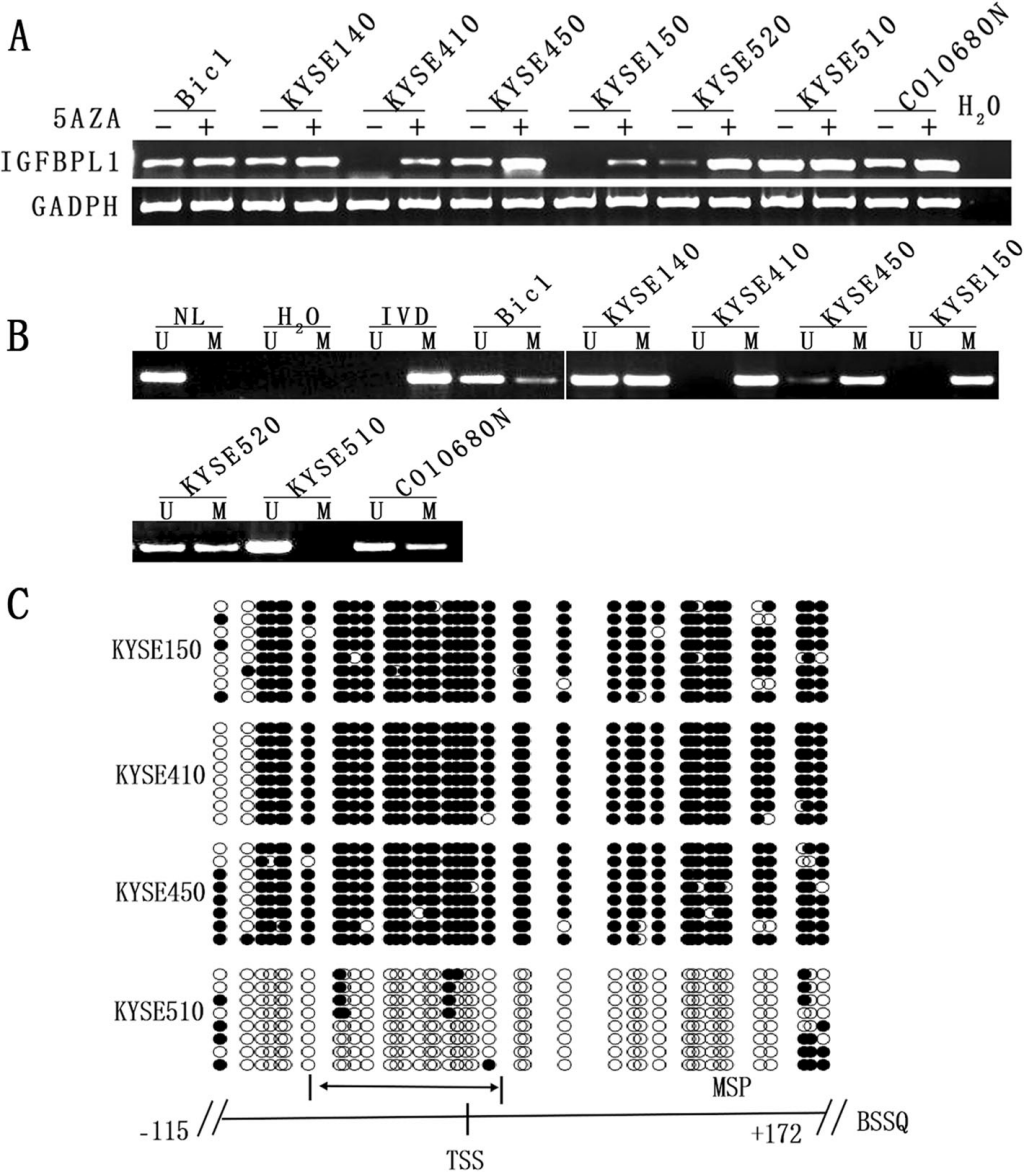
IGFBPL1 expression and methylation status in esophageal cancer cells. **a** The expression of IGFBPL1 was detected by semi-quantitative RT-PCR. H_2_O, negative control; GAPDH, internal control. 5-AZA, 5-AZA-2′-deoxycytidine. “−” indicates the absence of 5-AZA; “+” indicated the presence of 5-AZA. **b** MSP results of IGFBPL1 in esophageal cancer cell lines. IVD, in vitro-methylated DNA (methylation control); NL, normal lymphocyte DNA (unmethylation control); H_2_O, double distilled water; U, unmethylated alleles; M, methylated alleles. **c** Bisulfite sequencing results of IGFBPL1 in KYSE150, KYSE410, KYSE450, and KYSE510 cells. Double-headed arrow indicates MSP PCR product size was 98 bp and bisulfite sequencing focused on a 287 bp region of the CpG island (from − 115 to + 172) around the IGFBPL1 transcription start site. Filled circles indicate methylated CpG sites. Open circles indicate unmethylated CpG sites; TSS: transcription start site

IGFBPL1 expression was detected by semi-quantitative RT-PCR in esophageal cancer cell lines. Reduced expression of IGFBPL1 was observed in Bic1, KYSE140, KYSE450, KYSE520, and COLO680N cells. High-level expression of IGFBPL1 was detected in KYSE510 cells, while the expression of IGFBPL1 was absent in KYSE410 and KYSE150 cells (Fig. 1a).

Promoter region methylation status was detected by MSP. Unmethylation was found in KYSE510 cells, partial methylation was observed in Bic1, KYSE140, KYSE450, KYSE520, and COLO680N cells, and complete methylation was found in KYSE410 and KYSE150 cells (Fig. 1b). The expression of IGFBPL1 was inversely associated with promoter region methylation. To further reveal that the expression of IGFBPL1 is regulated by promoter methylation, cells were treated with 5-AZA. As expected, the levels of IGFBPL1 expression were unchanged in KYSE510 cells after 5-AZA treatment, while the expression of IGFBPL1 increased in Bic, KYSE140, KYSE450, KYSE520 and COLO680N cells, and the expression of IGFBPL1 was restored in KYSE410 and KYSE150 cells (Fig. 1a). To further validate the efficiency of MSP primers and explore the methylation density of esophageal squamous cancer cells, BSSQ technique was employed. As shown in Fig. 1c, IGFBPL1 was completely methylated in KYSE150 and KYSE410 cells, partially methylated in KYSE450 cells, and unmethylated in KYSE510 cells. These results were consistent with the MSP results. Taken together, these results suggested that the expression of IGFBPL1 is regulated by promoter region methylation in esophageal cancer cells.

### IGFBPL1 is frequently methylated in human esophageal dysplasia and ESSC

**Fig. 2:**
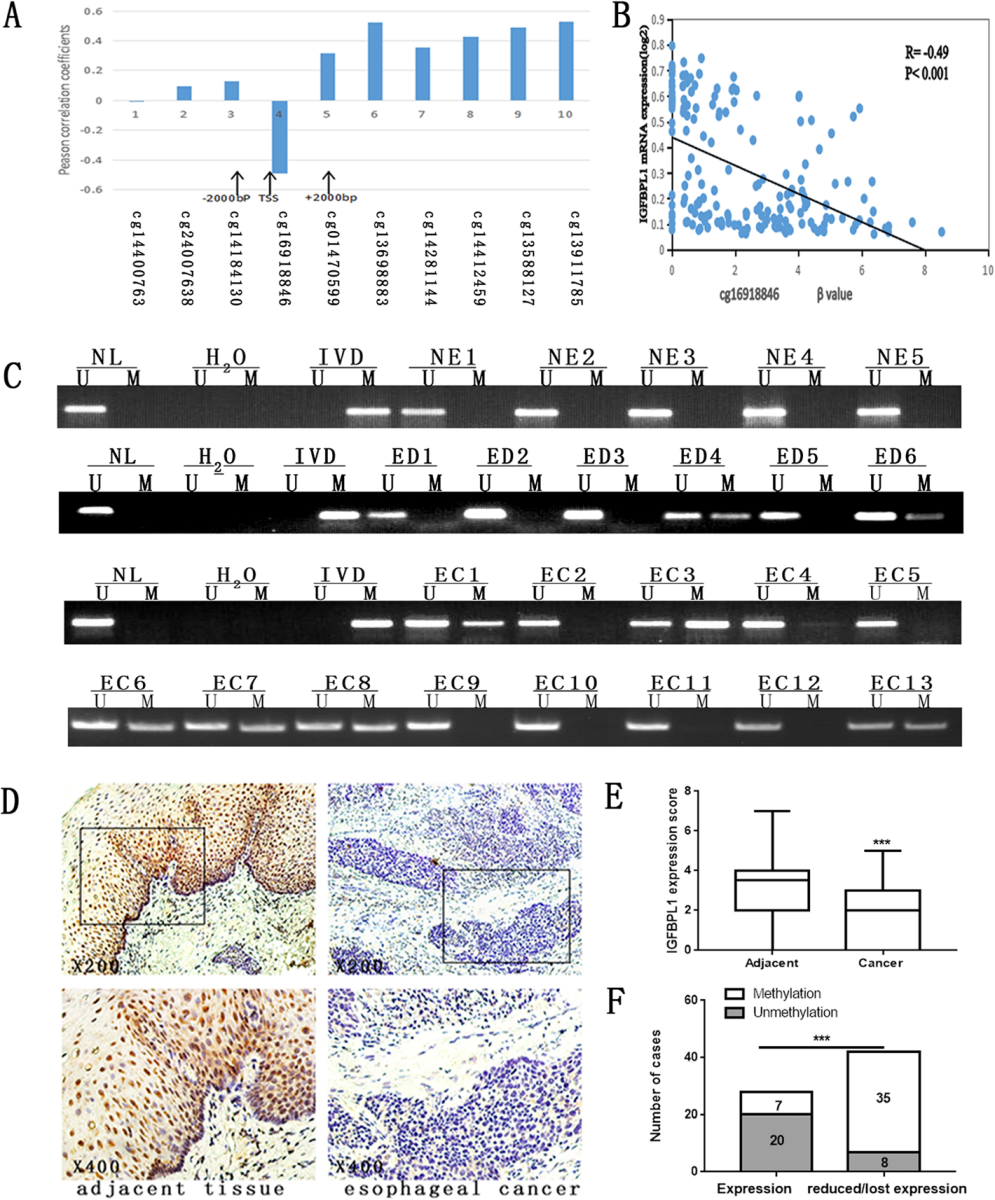
The expression and methylation status of IGFBPL1 in human esophageal dysplasia and ESCC. **a** Pearson correlation coefficient between IGFBPL1 methylation and expression of each CpG site. **b** Scatter plots showing the methylation status of the 4th (cg16918846) CpG sites, which are correlated with loss or reduced IGFBPL1 expression in 186 cases of ESCC tissue samples. ***p < 0.001. **c** Representative methylation results of IGFBPL1 in normal esophageal mucosa (NE), esophageal dysplasia (ED), and esophageal cancer (EC). The frequency of IGFBPL1 methylation was analyzed by chi-square test. **d** Representative IHC staining of IGFBPL1 in esophageal cancer (right panels) and adjacent tissue (left panels). Upper panels, × 200; lower panels, × 400. **e** The IGFBPL expression score is shown as a block diagram; the horizontal line represents the median score; the bottom and top of the box represent the 25th and 75th percentile, respectively; and the vertical bar chart indicates the scope of the data. There were significant differences in the expression of IGFBPL1 in adjacent tissues and cancer tissues in 70 cases of esophageal cancer. ***p < 0.001. **f** The expression of IGFBPL1 and DNA methylation status is shown as a bar diagram. Reduced or lost expression of IGFBPL1 was significantly associated with promoter region hypermethylation. ***p < 0.001

The Cancer Genome Atlas (TCGA) database was employed to predict whether the expression of IGFBPL1 is regulated by promoter region methylation. RNA expression and methylation data were extracted from the TCGA database (http://xena.ucsc.edu/) for 186 cases of esophageal primary cancer. IGFBPL1 methylation was only analyzed in 10 CpG sites. Reduced expression of IGFBPL1 was associated with cg-16,918,846 site methylation, which is located in the promoter region (Fig. 2a, b, *p* < 0.001). These results further indicated that the expression of IGFBPL1 is regulated by promoter region methylation.

IGFBPL1 methylation was detected by MSP in 114 cases of dysplasia, 501 cases of primary ESCC, and 5 cases of normal esophageal mucosa. IGFBPL1 was methylated in 47.3% (53/114) of esophageal dysplasia and 49.1% (246/501) of human primary ESCC, while no methylation was found in normal esophageal mucosa (Fig. 2c). Methylation of IGFBPL1 was significantly associated with TNM stage (*p* = 0.012) and tumor size (*p* = 0.009) (Table 1), whereas it was not associated with age, gender, lymph node metastasis, tumor cell differentiation, cancer embolus, distal metastasis, smoking, alcohol consumption, or family history (all *p* > 0.05). To further analyze whether IGFBPL1 expression is regulated by promoter region methylation, 70 cases of available matched esophageal cancer and adjacent tissue samples were evaluated by IHC analysis. IGFBPL1 staining was found in both the nucleus and cytoplasm of the esophagus. IGFBPL1 was highly expressed in the paired paracancerous tissues, while expression was reduced in cancer tissues (Fig. 2d, e, *p* < 0.001). The reduced expression of IGFBPL1 was significantly associated with promoter region hypermethylation (Fig. 2f, *p* < 0.001). These results further suggested that the expression of IGFBPL1 is regulated by promoter region methylation in human esophageal cancer.

**Table 1:** The association of IGFBPL1 methylation and clinical factors in human ESCC

Clinical factor	Number	IGFBPL1 methylation status		*P* value
		Unmethylated *n* = 255 50.28%	Methylated *n* = 246 49.10%	
Age (years)				0.167
< 63	252	136	116	
≥ 63	249	119	130	
Gender				0.102
Male	316	152	164	
Female	185	103	82	
Tumor size (cm)				0.009**
< 4	254	144	110	
≥ 4	247	111	136	
Differentiation				0.518
Well or moderate	390	200	190	
Poor	111	55	56	
Lymph node metastasis				0.131
Negative	310	166	144	
Positive	191	89	102	
TNM stage				0.012*
Stage I-II	295	164	131	
Stage III-VI	206	91	115	
Distal metastasis				0.070
Yes	58	36	22	
No	443	219	224	
Cancer embolus				0.848
Yes	15	8	7	
No	486	247	239	
Smoking				0.277
Yes	271	144	127	
No	230	111	119	
Drinking				0.136
Yes	116	52	64	
No	385	203	182	
Inherited				0.806
Yes	136	68	68	
No	365	187	178	

*P* values are obtained from chi-square test, significant difference

*p < 0.05

**p < 0.01

### IGFBPL1 inhibits esophageal cancer cell proliferation

**Fig. 3:**
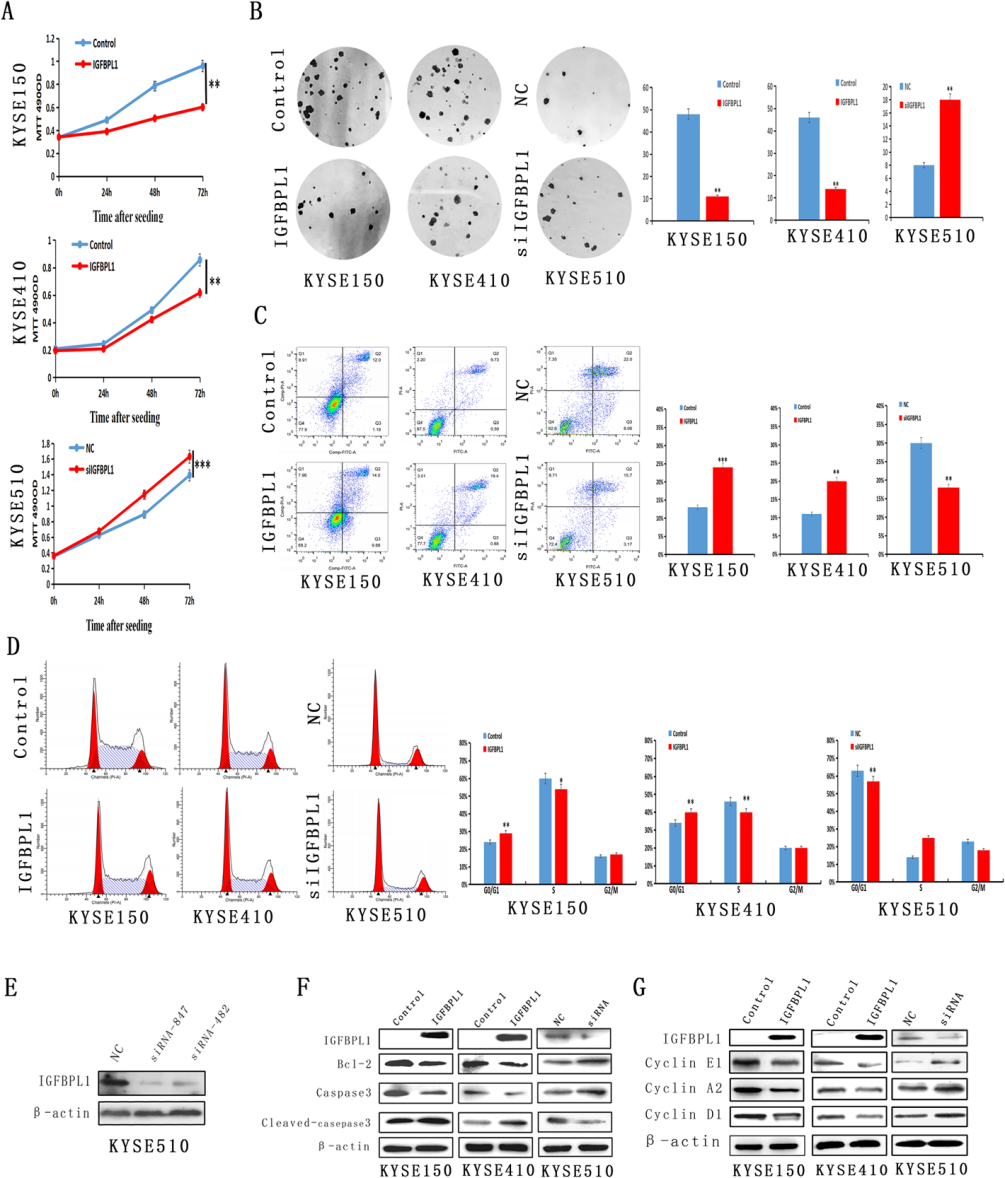
IGFBPL1 inhibits esophageal cancer cell proliferation and induces cell apoptosis and G1/S phase arrest. **a** The effect of IGFBPL1 on cell viability was examined by the MTT assay. The growth curves of IGFBPL1 re-expressed and unexpressed KYSE150 and KYSE410 cells, and KYSE510 cells before and after IGFBPL1 gene knockdown, were analyzed. Each experiment was repeated three times. **p < 0.01, ***p < 0.001. **b** Colony formation assays showed that the number of clones decreased after the expression of IGFBPL1 was restored in KYSE150 and KYSE410 cells, while the number of clones of KYSE510 cells increased after IGFBPL1 knockdown. Each experiment was repeated three times. **p < 0.01, ***p < 0.001. **c** Flow cytometry results showed that over-expression of IGFBPL1 in KYSE150 and KYSE410 cells induced apoptosis, whereas apoptosis decreased after knockdown of IGFBPL1 in KYSE510 cells. Each experiment was repeated three times. **p < 0.01, ***p < 0.001. **d** Cell cycle distribution of KYSE150 and KYSE410 cells that had no IGFBPL1 expression or re-expression of IGFBPL1 and KYSE510 before and after knockdown of IGFBPL1. Each experiment was repeated three times. **p < 0.0 1, ***p < 0.001. **e** IGFBPL1 in KYSE510 cells was knocked down by siRNA. IGFBPL1 expression was examined by Western blot. NC, siRNA for IGFBPL1 negative control; siRNA847 and siRNA482, siRNA for IGFBPL1. **f** Western blots showed that IGFBPL1 has an effect on the expression levels of caspase-3 and Bcl-2 in KYSE150, KYSE410, and KYSE510 cells. Control, control vector; IGFBPL1, IGFBPL1 expression vector; β-actin, internal control; NC, siRNA negative control; siRNA, siIGFBPL1. **g** Western blots validated the effect of IGFBPL1 on the protein expression levels of cyclin E1, cyclin A2, and cyclin D1. Control, control vector; IGFBPL1, IGFBPL1 expressing vector; β-actin, internal control; NC, siRNA negative control; siRNA, siIGFBPL1

To evaluate the effect of IGFBPL1 expression on cell growth, the MTT assay was employed. The OD values were 0.9607 ± 0.0603 vs. 0.6034 ± 0.0299 in KYSE150 cells (*t* test, *p* < 0.01) and 0.8575 ± 0.0820 vs. 0.6167 ± 0.0108 (*t* test, *p* < 0.01) in KYSE410 cells before and after the restoration of IGFBPL1 expression (Fig. 3a). The OD values were 1.3924 ± 0.0304 vs. 1.6345 ± 0.0652 (*t* test, *p* < 0.001) in KYSE510 before and after the knockdown of IGFBPL1 (Fig. 3a). These results demonstrated that IGFBPL1 inhibits esophageal cancer cell viability.

The effect of IGFBPL1 on cell proliferation was detected by colony formation assays. The clone numbers were 52 ± 1.4 vs.11 ± 1.1 in KYSE150 cells (*t* test, *p* < 0.01) and 47 ± 1.2 vs. 15 ± 1.1 in KYSE410 cells (*t* test, *p* < 0.01) before and after over-expression of IGFBPL1 (Fig. 3b). The clone numbers were 8 ± 1.3 vs.18 ± 3.0 (*t* test, *p* < 0.001) in KYSE510 cells before and after the knockdown of IGFBPL1 (Fig. 3b). These results suggested that IGFBPL1 inhibits esophageal cancer cell proliferation.

### IGFBPL1 induces esophageal cancer cell apoptosis

Flow cytometry was used to analyze the effect of IGFBPL1 on apoptosis. The ratios of apoptotic cells in IGFBPL1 unexpressed and re-expressed cells were 13.18 ± 0.18% vs. 23.88 ± 0.15% (*t* test, *p* < 0.001) in KYSE150 cells and 10.32 ± 0.17% vs.19.28 ± 0.14% (*t* test, *p* < 0.01) in KYSE410 cells (Fig. 3c). The proportion of apoptotic cells increased significantly after re-expression of IGFBPL1 in KYSE150 and KYSE410 cells. The percentage of apoptotic cells before and after knockdown of IGFBPL1 in KYSE510 cells was 30.06 ± 0.12% vs. 18.87 ± 0.14% (*t* test, *p* < 0.01, Fig. 3c). The percentage of apoptotic cells was significantly reduced after knockdown of IGFBPL1. To further verify the effect of IGFBPL1 on apoptosis of esophageal cancer cells, the expression levels of caspase-3, cleaved-caspase-3, and Bcl-2 in esophageal cancer cells were analyzed. As shown in Fig. 3f, the levels of caspase-3 decreased and the levels of cleaved-caspase-3 and Bcl-2 decreased after IGFBPL1 was re-expressed in KYSE150 and KYSE410 cells. Meanwhile, the levels of caspase-3 and Bcl-2 increased and the levels of cleaved-caspase-3 decreased after knockdown of the IGFBPL1 in KYSE510 cells (Fig. 3e, f). These results further suggested that IGFBPL1 induces apoptosis in esophageal cancer cells.

### IGFBPL1 induces G1/S phase arrest in esophageal cancer cells

The effect of IGFBPL1 on cell cycle was analyzed by flow cytometry. In KYSE150 cells, the cell phase distribution patterns before and after re-expression of IGFBPL1 were as follows: 24.74 ± 0.42% vs. 28.94 ± 0.6% in G0/G1 phase (*p* < 0.001), 59.25 ± 3.35% vs. 54.83 ± 0.33% in S phase (*p* < 0.05), and 15.67 ± 1.38% vs.16.22 ± 0.47% in G2/M phase (*p* > 0.05) (Fig. 3d). The percentage of cells in G0/G1 phase increased significantly, whereas the percentage of cells in S phase decreased significantly after re-expression of IGFBPL1 (Fig. 3d). The cell cycle distribution patterns before and after re-expression of IGFBPL1 in KYSE410 cells were 34.57 ± 0.87% vs. 39.58 ± 0.59% in G0/G1 phase (*p* < 0.01), 45.74 ± 2.32% vs. 40.82 ± 0.27% in S phase (*p* < 0.05), and 19.28 ± 0.85% vs.19.36 ± 1.17% in G2/M phase (*p* > 0.05, Fig. 3d). The G0/G1 phase cells increased significantly, whereas the S phase cells decreased significantly after re-expression of IGFBPL1 in KYSE410 cells (Fig. 3d). The effect of IGFBPL1 on cell cycle was further validated by knocking down IGFBPL1 in IGFBPL1 highly expressed KYSE510 cells. The distribution of cell phases were 62.29 ± 0.22% vs. 57.38 ± 0.68% in G0/G1 phase (*p* < 0.01), 18.89 ± 1.12% vs. 26.11 ± 0.97% in S phase (*p* > 0.05), and 18.82 ± 0.58% vs. 16.35 ± 1.02% in G2/M phase (*p* > 0.05, Fig. 3d). The distribution of G0/G1 phase increased significantly after knockdown of IGFBPL1 (Fig. 3d). To further validate the effect of IGFBPL1 on cell cycle, the protein expression levels of cyclinE1, cyclinA2, and cyclinD1 were detected in KYSE150 and KYSE410 cells by Western blot before and after re-expression of IGFBPL1. As shown in Fig. 3g, the levels of cyclinE1, cyclinA2, and cyclinD1 decreased after re-expression of IGFBPL1 in KYSE150 and KYSE410 cells. The effect of knocking down IGFBPL1 on cyclinE1, cyclinA2, and cyclinD1 expression was verified in KYSE510 cells as well. The levels of cyclinE1, cyclinA2, and cyclinD1 increased after knockdown of IGFBPL1 (Fig. 3g). These results indicated that IGFBPL1 induces G1/S phase arrest in human esophageal cancer cells.

### IGFBPL1 inhibits PI3K-AKT signaling in human esophageal cancer cells

**Fig. 4:**
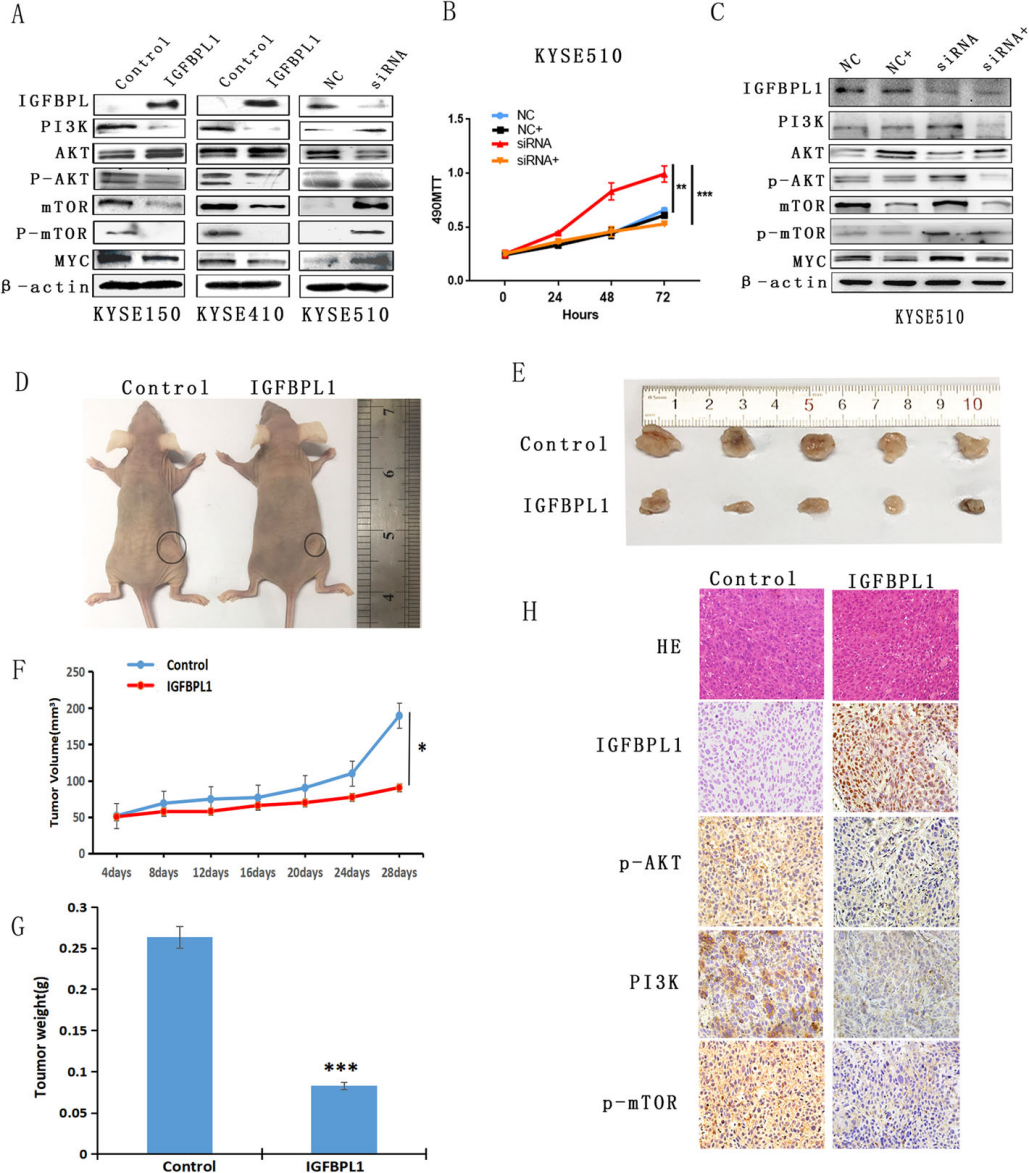
IGFBPL1 inhibits the PI3K-AKT signaling pathway and suppresses human ESCC cell xenograft growth in mice. **a** Western blots showed that IGFBPL1 has an effect on the expression levels of PI3K, AKT, p-AKT, mTOR, p-mTOR, and MYC in KYSE150, KYSE410, and KYSE510 cells. Control, control vector; IGFBPL1, IGFBPL1 expression vector; β-actin, internal control; NC, siRNA negative control; siRNA, siIGFBPL1. **b** Growth curves represent cell viability evaluated by MTT assay in the control group, control plus NVP-BEZ235(100 nM) treatment group, siIGFBPL1 group, and siIGFBPL1 plus NVP-BEZ235 treatment group in KYSE510 cells. NC, control group; NC+, control plus NVP-BEZ235 treatment group; siRNA, siIGFBPL1; siRNA+, siIGFBPL1 plus NVP-BEZ235 treatment group. **p < 0.0 1, ***p < 0.001. **c** Western blots showed that IGFBPL1 has an effect on the expression levels of PI3K, AKT, p-AKT, mTOR, p-mTOR, and MYC in KYSE510 cells before and after NVP-BEZ235 treatment. NC, control group; NC+, control plus NVP-BEZ235 treatment group; siRNA, siIGFBPL1; siRNA+, siIGFBPL1 plus NVP-BEZ235 treatment group. **d**, **e** Represents tumors from KYSE150 cell xenografts in which IGFBPL1 is not expressed and IGFBPL1 is over-expressed. **f** Tumor growth curves of unexpressed IGFBPL1 and IGFBPL1 overexpressing KYSE150 cells. *p < 0.05. **g** Tumor weight at 28th day after inoculation of unexpressed IGFBPL1 and IGFBPL1 overexpressing KYSE150 cells in nude mice. Bars indicate mean of five mice. ***p < 0.001. **h** Images of hematoxylin and eosin staining show tumors from IGFBPL1 unexpressed and IGFBPL1 re-expressed KYSE150 xenograft mice. IHC staining reveals the expression levels of IGFBPL1, p-AKT, PI3K, and p-mTOR in IGFBPL1 unexpressed and IGFBPL1 re-expressed KYSE150 cell xenografts. Magnification, × 400

IGFBPL1 has been reported to be involved in PI3K-AKT signaling during neural development in mice [15]. To further understand the mechanism of IGFBPL1 in esophageal cancer development, we studied the role of IGFBPL1 in PI3K-AKT signaling in esophageal cancer cells. As shown in Fig. 4a, the levels of PI3K, p-AKT, mTOR, p-mTOR, and MYC were decreased and the levels of AKT were increased after re-expression of IGFBPL1 in KYSE150 and KYSE410 cells, while the levels of PI3K, p-AKT, mTOR, p-mTOR, and MYC were increased and the levels of AKT were reduced after knockdown of IGFBPL1 in KYSE510 cells. These results suggested that IGFBPL1 inhibits PI3K-AKT signaling in esophageal cancer cells.

To further validate the effect of IGFBPL1 on PI3K-mTOR signaling, NVP-BEZ235, a PI3K-mTOR inhibitor, was employed, and the MTT assay was used to evaluate cell proliferation ability. The OD values in KYSE510 cells were 0.658 ± 0.021 in the control group, 0.611 ± 0.016 in control plus NVP-BEZ235 treatment group, 0.993 ± 0.022 in siIGFBPL1 group, and 0.529 ± 0.014 in siIGFBPL1 plus NVP-BEZ235 treatment group. No significant differences were found between the control group and control plus NVP-BEZ235 treatment group, as well as between the control group and siIGFBPL1 plus NVP-BEZ235 treatment group (both *p* > 0.05) in IGFBPL1 highly expressed KYSE510 cells. However, the OD value increased significantly in the siIGFBPL1 group compared to the control group (*p* < 0.01 Fig. 4b), and the OD value decreased significantly in siIGFBPL1 plus NVP-BEZ235 group compared to siIGFBPL1 group (*p* < 0.01, Fig. 4b). These results further suggested that IGFBPL1 inhibits PI3K signaling in esophageal cancer cells.

To further verify the effect of IGFBPL1 on PI3K-AKT signaling, the levels of PI3K, AKT, p-AKT, mTOR, p-mTOR, and MYC expression were detected by Western blot in IGFBPL1 highly expressed KYSE510 cells with or without NVP-BEZ235 treatment, as well as in siIGFBPL1-treated KYSE510 cells. The levels of PI3K, p-AKT, mTOR, p-mTOR, and MYC were decreased after NVP-BEZ235 treatment in KYSE510 cells. The levels of PI3K, p-AKT, mTOR, p-mTOR, and MYC were increased after siIGFBPL1 knockdown KYSE510 cells (Fig. 4c). The levels of PI3K, p-AKT, mTOR, p-mTOR, and MYC were reduced after NVP-BEZ235 treatment in siIGFBPL1 KYSE510 cells (Fig. 4c). These results further suggested that IGFBPL1 is involved in PI3K-AKT signaling in human ESCC.

### IGFBPL1 suppresses human esophageal cancer cell xenografts growth by inhibiting PI3K-AKT signaling

To further validate the effect of IGFBPL1 in ESCC, an esophageal cancer cell xenograft mouse model was employed (Fig. 4d). KYSE150 cells before and after re-expression of IGFBPL1 were inoculated subcutaneously in nude mice. The mean tumor volume was 189.53 ± 18.42 mm^3^ and 90.54 ± 17.29 mm^3^ in IGFBPL1 unexpressed and re-expressed KYSE150 cell xenografts, respectively. The xenograft volume decreased significantly after re-expression of IGFBPL1 in KYSE150 cells (*t* test, *p* < 0.05, Fig. 4e, f). The tumor weight was 265.58 ± 46.86 mg vs. 83.08 ± 18.12 mg in IGFBPL1 unexpressed and re-expressed KYSE150 cell xenografts. The tumor weight decreased significantly in IGFBPL1 re-expressed KYSE150 cell xenografts (*t* test, *p* < 0.001, Fig. 4g). The expression of IGFBPL1 was validated in IGFBPL1 re-expressed KYSE150 cell xenografts by IHC staining (Fig. 4h, lower panels). These results indicated that IGFBPL1 suppresses esophageal cancer cell growth in vivo.

To further validate whether IGFBPL1 inhibits PI3K-AKT signaling in vivo, the levels of PI3K, p-AKT, and p-mTOR were detected by IHC staining before and after re-expression of IGFBPL1 in KYSE150 cell xenografts. The expression of PI3K, p-AKT, and p-mTOR decreased in IGFBPL1 re-expressed KYSE150 cell xenografts (Fig. 4h). These results suggested that IGFBPL1 inhibits PI3K-AKT signaling in vivo.

## Discussion

Insulin-like growth factor (IGF) signaling plays important roles in regulating growth and development in normal human tissues by promoting cellular proliferation and differentiation and preventing apoptosis [24, 25]. IGF-1 and IGF-2 are members of the insulin superfamily of growth-promoting peptides and are among the most abundant and ubiquitous polypeptide growth factors [26]. Increased levels of IGF-1 and IGF-2 are associated with many cancers, including esophageal cancer [27–30].

The IGFs are distinguished from insulin by their interaction with six high-affinity IGFBPs [31]. In addition to modulating IGF bioactivity, IGFBP family members have biological actions independent of their abilities to bind IGFs, including binding to a variety of extracellular and cell surface molecules [27]. IGFBPs are cysteine-rich proteins that bind to IGFs with high affinity, thereby enhancing or inhibiting the IGF signaling pathway in a cell-type-dependent manner [15]. Increased expression of IGF1R, IGFBP3, IGFBP4, IGFBP7, and IGFBP8 was reported in human esophageal cancer, while the expression of IGFBP2 and IGFBP6 was reduced [32–35]. While the levels of IGFBP1-6 mRNA expression in TCGA and GTEx database are different with other reports, the expression of IGFBP1 and IGFBP3 were increased in ESCC compared to normal tissue samples (all *p* < 0.001). The expression of IGFBP5 and IGFBP6 were reduced in ESCC compared to normal tissue samples (all *p* < 0.001). No significant expression changes were found in IGFBP2 and IGFBP4 in ESCC and normal tissue samples (all *p* > 0.05). No association was found between IGFBPs expression and overall survival in ESCC patients (all *p* > 0.05, data not shown). We further evaluated IGFBP1-6 expression in esophageal cancer cells by RT-PCR. Loss of IGFBP1-5 expression was frequently found in esophageal cancer cells, while IGFBP6 was expressed in all cell lines (data not show). To date, no cancers have been attributed to IGFBP mutation [36]. IGFBPL1 was identified in 1997 (Fujimoto, GenBank Submission, 1997) and is located on chromosome 4 in mouse and chromosome 9p13.1 in humans. The IGFBPL1 gene was reported to be frequently methylated in human breast cancer and regulated by promoter region methylation, and methylation of IGFBPL1 was associated with poor prognosis [17].

In this study, we found that the expression of IGFBPL1 is regulated by promoter region methylation in human esophageal cancer. IGFBPL1 is frequently methylated in human esophageal dysplasia and ESCC. Methylation of IGFBPL1 is associated with tumor size and TNM stage and therefore may serve as an esophageal cancer early detection marker. Further study found that IGFBPL1 suppresses esophageal cancer cell growth both in vitro and in vivo. IGFBPL1 was reported to initiate axon growth by phosphorylation of PI3K and mTOR during development [16, 37–39]. Then, we analyzed the effect of IGFBPL1 on PI3K-AKT pathway in esophageal cancer. Our results demonstrate that IGFBPL1 inhibited PI3K-AKT signaling in human esophageal cancer cells. Thus, methylation of IGFBPL1 may activate PI3K-AKT signaling in ESCC. These results suggest IGFBPL1 methylation may serve as a predictive marker for PI3K-targeted therapy in ESCC.

## Conclusion

IGFBPL1 is frequently methylated in human esophageal dysplasia and ESCC, and its expression is regulated by promoter region methylation. Methylation of IGFBPL1 is associated with tumor size and TNM stage. IGFBPL1 suppresses esophageal cancer cell growth by inhibiting PI3K-AKT signaling in vitro and in vivo. Methylation of IGFBPL1 is a potential esophageal cancer early detection marker and a predictive marker for PI3K-targeted therapy in ESCC.

## Data Availability

Based on a reasonable request, the data from the current research analysis can be obtained from the corresponding author.

## Abbreviations

5-AZA: 5-AZA-2′-deoxycytidine
BSSQ: Bisulfite sequencing
EAC: Esophageal adenocarcinoma
ESCC: Esophageal squamous cell carcinoma
GAPDH: Glyceraldehyde-3-phosphate dehydrogenase
IHC: Immunohistochemistry
IVD: In vitro-methylated DNA
MSP: Methylation-specific PCR
NL: Normal lymphocyte DNA
TCGA: The Cancer Genome Atlas
TSS: Transcription start site
